# Japan’s voluntary lockdown: further evidence based on age-specific mobile location data

**DOI:** 10.1007/s42973-021-00077-9

**Published:** 2021-06-21

**Authors:** Tsutomu Watanabe, Tomoyoshi Yabu

**Affiliations:** 1 Graduate School of Economics, University of Tokyo, Tokyo, Japan; 2 Faculty of Business and Commerce, Keio University, Tokyo, Japan

**Keywords:** Covid-19, Pandemic, Social behavior change, Stay at home, Lockdown, State of emergency, Age-specific mobile location data, E60, H12, H70, I12, L51

## Abstract

Changes in people’s behavior during the COVID-19 pandemic can be regarded as the result of two types of effects: the “intervention effect” (changes resulting from government orders for people to change their behavior) and the “information effect” (voluntary changes in people’s behavior based on information about the pandemic). Using age-specific mobile location data, we examine how the intervention and information effects differ across age groups. Our main findings are as follows. First, the age profile of the intervention effect shows that the degree to which people refrained from going out was smaller for older age groups, who are at a higher risk of serious illness and death, than for younger age groups. Second, the age profile of the information effect shows that the degree to which people stayed at home tended to increase with age for weekends and holidays. Thus, while Acemoglu et al. (2020) proposed targeted lockdowns requiring stricter lockdown policies for the oldest group in order to protect those at a high risk of serious illness and death, our findings suggest that Japan’s government intervention had a very different effect in that it primarily reduced outings by the young, and what led to the quarantining of older groups at higher risk instead was people’s voluntary response to information about the pandemic. Third, the information effect has been on a downward trend since the summer of 2020. It is relatively more pronounced among the young, so that the age profile of the information effect remains upward sloping.

## Introduction

The number of COVID-19 infections in Japan began to increase in earnest in the latter half of February, and by the end of March, the cumulative number of infections had reached 2234. In response to the spread of infections, the government declared a state of emergency on April 7 for seven prefectures including Tokyo, and on April 16, the state of emergency was expanded to cover all prefectures. As a result, people refrained from going out, and the number of new infections in Japan, after peaking at 720 on April 11, began to drop, falling to almost zero by the end of May. This was the first wave of infections. However, in July, the number of new infections began to increase again, and continued to increase throughout the summer (peaking at 1605 new infections on August 7). This was the second wave. While the second wave had subsided by the end of August, the number of new infections began to increase once again in late October, and on December 31, 2020, the number of new infections in Tokyo reached 1353, exceeding 1,000 for the first time (the number of new infections nationwide was 4534). In response, the government again declared a state of emergency on January 7. We are currently in the middle of the third wave.

The increase and decline in infections are closely linked to people’s behavior in terms of leaving their homes. Previous research on changes in behavior has focused on two channels. The first channel is called the “intervention effect,” which refers to changes in behavior as a result of government orders or requests. In China, the United Sates, and European countries, governments have declared lockdowns when infection rose, issuing “orders” for people to stay at home. In contrast, unlike the lockdowns in these countries, restrictions during Japan’s state of emergency had no legal binding force, and there were no fines, arrests, or other punishments imposed for leaving home during the state of emergency. The Japanese government only verbally “requested” people to refrain from going out, and many people heeded this “request” by staying at home. In this sense, Japan’s declaration of a state of emergency can be regarded as a “voluntary lockdown.” The second channel is the “information effect,” which refers to voluntary changes in people’s behavior based on information about the pandemic (such as the number of new infections, number of deaths, etc.). There are various possible sources of information that people can rely on. However, in developed countries including Japan, details on infections are rarely disclosed to the public in order to protect the privacy of those infected. This means that governments have considerably more information than is in the public domain (at least this is what many people believe), so that information transmitted by the government is likely to have a greater impact than in normal times.

In an earlier study (Watanabe & Yabu, 2020), we estimated the two effects using data for Japan during the first wave and found that the government intervention of declaring a state of emergency had the effect of reducing outings by 8.5% compared to before the coronavirus period. On the other hand, estimating the two effects using data for the United States, Goolsbee & Syverson (2021) found that the intervention effect associated with shelter-in-place (S-I-P) orders in the United States was 7.6%. What is interesting is that the size of the estimated intervention effects is of the same order of magnitude, despite the fact that the measures in Japan and the United States differed substantially in terms of whether they were legally binding or not. However, while we interpreted our estimate for Japan as showing that Japan’s voluntary lockdown had a reasonably large effect despite the fact that the measures were not legally binding, Goolsbee& Syverson (2021) judged the effect of the S-I-P orders in the United States to have been limited despite the fact that the measures were strictly legally binding. Thus, even though the size of the effect was very similar, our assessment and theirs were very different.

In this study, we elaborate on the analysis in Watanabe & Yabu (2020) by extending the observation period to include the second and third waves of the pandemic in Japan, creating stay-at-home measures for different age groups, and estimating the intervention and information effects for each age group. The main purpose of our analysis is to determine how the intervention and information effects differ (or do not differ) by age. This matters because the serious illness rate and infection fatality rate for those infected with COVID-19 vary greatly with age. According to figures published by the Japanese government (MHLW, 2020), the serious illness rate is 150 times higher for people in their 70s than for those in their 20s, an extremely large difference. Large differences in the serious illness and infection fatality rates by age group have also been reported for other countries, and substantial differences in the way that COVID-19 affects different age groups is one of the most important characteristics of the pandemic. Focusing on these differences, Acemoglu et al. (2020) argue that targeted lockdowns for older age groups, rather than uniform lockdowns for all age groups, would be more desirable in terms of effectively controlling infections while reducing the impact on economic activity. Similar arguments have been made by medical researchers (e.g., Smith & Spiegelhalter, 2020). In this study, we extend the methodology presented in Watanabe & Yabu (2020) and use it to estimate the effect of interventions by age group. This allows us to examine whether the Japanese government’s intervention policies, such as the declaration of a state of emergency and the closure of schools, meet the criteria of optimal intervention policies highlighted by Acemoglu et al. (2020), i.e., policies that maximize protection for those at greatest risk while minimizing the impact on economic activity. This is the first objective of this study.

Our earlier study, Watanabe & Yabu (2020), showed that only one quarter of the reduction in outings in Tokyo during the first wave can be explained by government interventions, while the remaining three quarters were the result of people voluntarily changing their behavior as a result of information about the pandemic. In this study, we examine the “information effect” in more detail. Given that the information effect reflects information about COVID-19 infections and deaths, it to a considerable extent reflects the impact of fear on people’s behavior, and numerous studies have focused on this “fear effect.” For instance, Cochrane (2020) incorporates into the canonical epidemiological model, the SIR model, an equation in which the transmission rate of the virus depends on the number of new infections and deaths, based on the assumption that people decide whether to go out depending on the number of new infections and deaths announced daily. Aum et al. (2020a) develop a setting in which fear of infection leads to disutility. Solving their optimization problem under this setting, people choose not to go out or to work from home. The aspect of fear of COVID-19 has also featured prominently in the field of psychology, where researchers have created a “Fear of COVID-19 Scale” (Ahorsu et al., 2020)^1^ and, by investigating in experiments whether such indicators predict changes in behavior (such as hand washing, social distancing, stockpiling food, working from home), have shown that fear is the most important predictor of changes in behavior (Harper et al., 2020). In this study, we follow this line of research and assume that the information effect to a considerable extent reflects the “fear effect,” that is, the effect that people’s fear of infection increases as a result of exposure to information about the pandemic (such as an increase in the number of newly infected people), which in turn leads them to refrain from going out. An important implication of the fear effect defined in this way is that it is age dependent. In other words, even if older and younger age groups are exposed to the same information about infections, their level of fear will be different, and so will be the degree to which they refrain from going out. The second aim of this study is to test this implication of the information effect by estimating the information effect for each age group.

For our analysis, we use smartphone location data to construct a daily measure by prefecture, gender, and age showing the degree to which people stayed at home. We then construct panel data, which we use to distinguish between the intervention and information effects. For the intervention effect, we use the variation in the stay-at-home measure *across prefectures* for identification. For example, when the first state of emergency was declared on April 7, this covered only seven prefectures, including Tokyo, but not the other prefectures. Therefore, comparing the stay-at-home measures for prefecture A included the state of emergency and excluded prefecture B provides us with information about the intervention effect of the emergency declaration. When doing so, it is important to compare the same age group in both prefectures. For example, comparing the old in prefecture A with the old in prefecture B means that there will be no difference in the information effect because we are comparing people with the same risk of serious illness and death. This procedure therefore allows us to extract the intervention effect only. Next, the fear effect can be extracted using the variation in the stay-at-home measure *across age groups*. For example, if the number of new infections in Tokyo increased significantly on a certain day, we compare how the stay-at-home measures for those in their 20s and 70s living in Tokyo responded to the increase. While these people live in the same area (Tokyo in this case) and therefore have the same information about the pandemic (the number of new infections in this example), their risk of serious illness and death differs, so that their level of fear will also differ. Thus, by looking at the difference in the stay-at-home measure between those in their 20s and those in their 70s, we can identify the fear effect.

The main findings of this study are as follows. First, the age profile of the intervention effect of the emergency declarations issued in April and May 2020 shows that the degree to which older age groups with a higher risk of serious illness and death refrained from outings was smaller than that for younger age groups. This result indicates that the emergency declaration had the exact opposite effect of the targeted lockdowns proposed by Acemoglu et al. (2020), which aim to quarantine the old while allowing the young to carry on with economic activities, and may not have had the intended impact in terms of effectively minimizing both the public health consequences and economic losses.

Second, the age profile of the information effect shows that, unlike the intervention effect, the degree to which people stayed at home tended to increase with age for weekends and public holidays. This result suggests that people update their information about the pandemic daily and make decisions to refrain from outings based on their own risk of serious illness and death. In other words, what achieved a “targeted lockdown” à la Acemoglu et al. (2020) was not government interventions such as the declaration of a state of emergency but people’s voluntary behavior.

Third, the information effect has been on a downward trend since the summer of 2020. While this trend applies to all age groups, it is relatively more pronounced among the young, so that the age profile of the information effect remains upward sloping, suggesting that people’s response to information about the pandemic is commensurate with their risk of serious illness and death.

The remainder of this study is organized as follows. Sect. 2 provides an overview of the existing literature on the economic impact of the COVID-19 pandemic. Next, Sect. 3 presents a brief overview of the spread of COVID-19 in Japan. Sects. 4 and 5 then respectively describe the methodology and data used in the empirical analysis of this study, while Sect. 6 presents the empirical results. Finally, Sect. 7 summarizes the findings and discusses their policy implications.

## Literature review

A large number of studies on the economic impact of the COVID-19 pandemic have already been published, and the number keeps growing rapidly. Against this background, the present study is closely related to research in the following three areas.

The first is research on the effect of lockdown policies on the extent to which people go out or remain economically active. Studies focusing on the United States include (Forsythe et al., 2020; Rojas et al., 2020; Coibion et al., 2020; Goolsbee & Syverson, 2021; Alexander & Karger, 2020; and Gupta et al., 2020). For instance, taking advantage of the fact that the lockdowns across the United States have not occurred simultaneously but have been implemented at different times in different states and counties, Goolsbee and Syverson (2021) compare consumer traffic in counties under lockdown and counties not under lockdown at a particular point in time to find that the differences were not very large. Meanwhile, Rojas et al. (2020), focusing on the fact that the timing of school closures has varied across states, examine whether there was a difference in the number of new claims for unemployment insurance between states with and without school closures at a given point in time. They found no statistically significant difference. A similar result was also reported by Chetty et al. (2020). These studies suggest that the changes in behavior in the United States are not the result of legally binding government measures but rather the result of people’s voluntary response to the pandemic.

Turning to a similar study using data other than for the United States, Sheridan et al. (2020) compare Denmark, where the government imposed legal restrictions on outings and economic activity to prevent the spread of infections, with Sweden, where there was no such government intervention. Finding that the decline in economic activity in the two countries was quite similar, they argue that government intervention was not the main reason for the decline in economic activity. Chen et al. (2020), using data for Europe, and Aum et al. (2020b), using data for Korea, reach similar conclusions. Moreover, in our previous study (Watanabe & Yabu, 2020) using the same data as in this study, we found that the direct effect of Japan’s emergency declaration was limited.

The second area to which our study is related is research on optimal non-pharmaceutical interventions, given that the risk of severe illness and death from COVID-19 differs substantially across age groups. Acemoglu et al. (2020) extended the SIR model to a setting with multiple groups at different levels of risk of serious illness and death, and quantitatively examine optimal policies in this setting. They show that targeted lockdowns aimed at the old, who have a higher risk of severe illness and death, are more effective in minimizing both deaths and economic losses. The reason is that in targeted lockdowns infections can be restrained by imposing stricter lockdown policies on the old than in uniform lockdowns, which allows a commensurate loosening of restrictions on the young, reducing economic losses. Other analyses based on theoretical models taking the fact that the risk of serious illness and death differs substantially across age groups into account include (Baqaee et al., 2020; Brotherhood et al., 2020; Glover et al., 2020; Gollier, 2020). The idea of shielding people according to their risk (“stratify and shield” strategies) rather than shielding everyone uniformly has also been proposed in the field of medicine (see, e.g., Smith & Spiegelhalter, 2020).

The third area to which our study is related is empirical research on heterogeneity in changes in people’s behavior during the pandemic and the causes of such heterogeneity. Focusing on Italy, Portugal, and Spain, Caselli et al. (2020) use mobile phone location data by gender and age to examine the impact of lockdowns on people’s mobility in the three countries. They find that the mobility of women decreased more than that of men as a result of lockdowns and that the mobility of younger cohorts declined more than that of older cohorts. Possible reasons for the greater decline in mobility of the young cited by Caselli et al. (2020) include that the young may be more concerned about the health risks posed by COVID-19 than older cohorts, and that the young lost their jobs due to the closure of restaurants and other workplaces where many young people work. Meanwhile, Andersen et al. (2020) use bank account transaction data to examine how people’s responses to government intervention policies differ by age. Specifically, focusing on Denmark, where strict intervention policies were implemented, and Sweden, where no such policies were implemented, they compare spending by age group. The results show that the spending of the youngest group fell more in Denmark, where the government shut down restaurants, schools, etc., in March 2020, than in Sweden, while spending of the oldest group fell less in Denmark than in Sweden. The authors interpret their results regarding the heterogeneity in behavior between the young and the old as suggesting that the closure of restaurants and other facilities constrained the spending choices of individuals with low health risk (the young), while the social distancing laws created a safer environment for those with high health risk (i.e., older groups).

The heterogeneity in changes in behavior has also been studied using surveys. For instance, in a survey of 1500 Americans, Bordalo et al. (2020) found that risk perceptions with regard to COVID-19 differed significantly across age groups. Specifically, younger age groups tended to perceive the risks related to COVID-19 (risk of infection, risk of serious illness if infected, and risk of death if infected) as higher than older age groups. Moreover, reflecting the differences in risk perceptions, younger age groups changed their behavior, such as refraining from going out, to a stronger degree. Belot et al. (2020), based on surveys conducted in six countries including the United States, Japan, and China, show that negative non-financial effects of the crisis have been more pronounced among younger age groups and do not differ across income groups. Using survey data on individuals living in the United States, Papageorge et al. (2021) find that higher income was associated with higher levels of self-protective behaviors such as hand washing, mask wearing, and social distancing. Reasons, they suggest, include that those on higher incomes are more likely to be able to work from home and to have transitioned to teleworking.

## Outbreak of COVID-19 and policy responses in Japan

The first reported case of a COVID-19 infection in Japan—of a man who had traveled to Wuhan, China—was on January 15, 2020. Then, on February 5, ten passengers of a cruise ship docked at Yokohama Port were confirmed to have caught the virus. The first death in Japan was reported on February 13. Infections in Japan began to rise in earnest from the second half of February, and as of February 29, the cumulative number of infections had reached 242. Infections further accelerated in early March, so that by the end of the month the cumulative number of infections had reached 2234. In response to the spread of infections, the government on February 27 requested elementary, junior high, and high schools nationwide to temporarily close, and on March 24 decided to postpone the Tokyo Olympic Games scheduled for the summer of 2020. Furthermore, on April 7, a state of emergency was declared for seven prefectures including Tokyo, and on April 16, this was expanded to all prefectures. This was followed by the second wave in the summer of 2020, and the third wave has been underway since November 2020. On January 7, 2021, the government declared a state of emergency for the second time covering four prefectures including Tokyo, and on January 13, seven more prefectures including Osaka were added to the prefectures covered.

**Fig. 1:**
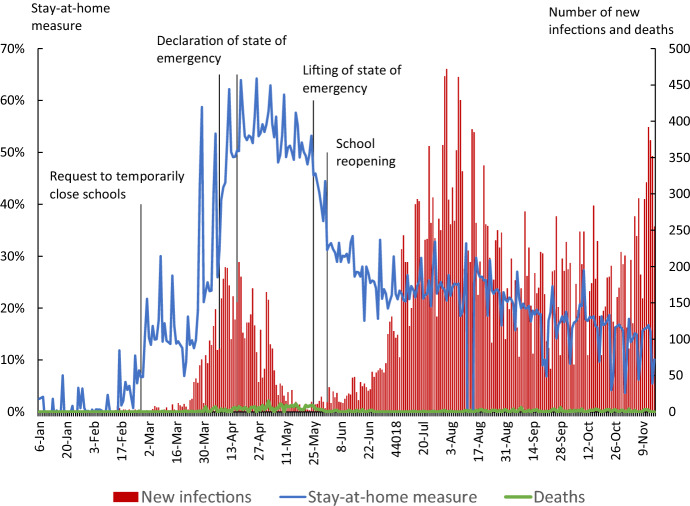
Stay-at-Home Measure and Number of New Infections and Deaths in Tokyo

Figure 1 shows the number of daily new infections in Tokyo, represented by the red bars. The number of new infections increased rapidly in late March, exceeding 100 on April 4 and exceeding 200 on April 17. With the declaration of the state of emergency, the number of new infections decreased and fell to almost zero in mid-May. This was the first wave of the pandemic. However, the number of new infections in Tokyo began to increase again in late June and continued to increase throughout the summer. This was the second wave. The number of new infections in Tokyo reached a peak of 472 on August 1. In November, the number of new infections began to increase again, reaching 486 on November 18, exceeding the peak of the second wave. The number of new infections has continued to rise since then, and we are currently in the middle of the third wave.

**Fig. 2:**
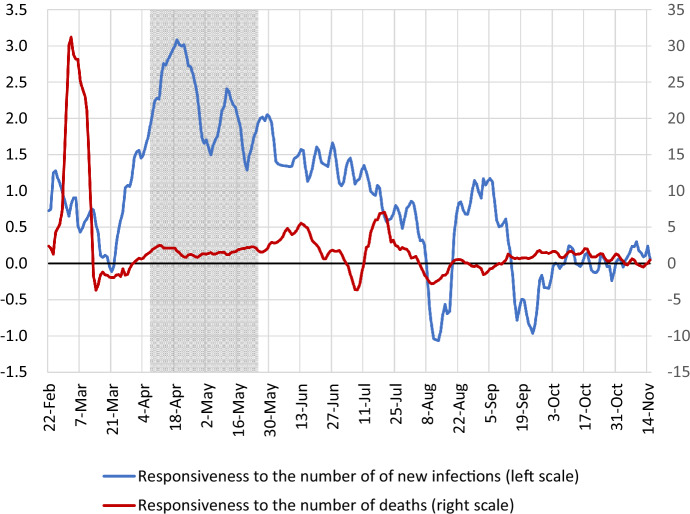
Responsiveness of Stay-at-Home Measure to Number of New Infections and Number of Deaths. Note: The figure shows the coefficients for the number of new infections and the number of deaths obtained using specification (4) in Table 1. The lines depict the centered 11-day moving averages of the coefficient estimates. The figure only shows the period from February 22, 2020, onward, when the cumulative number of infections surpassed 100. The shaded area represents the period of the state of emergency (April 7 to May 25, 2020)

**Fig. 3:**
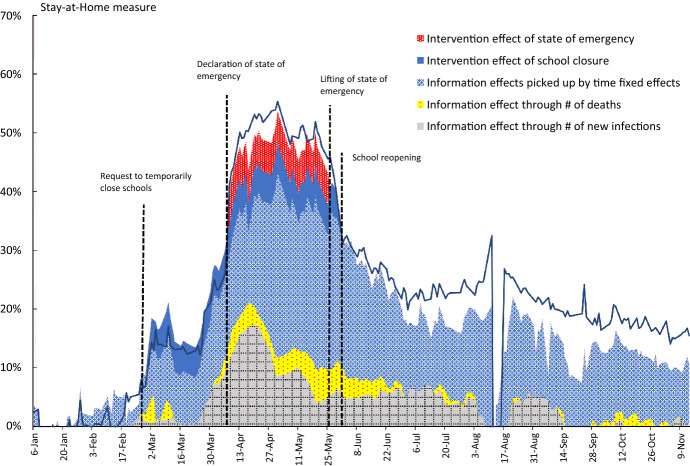
Decomposition of Changes in the Stay-at-Home Measure for Tokyo. Notes: Changes in the stay-at-home measure for Tokyo are decomposed into the intervention and the information effects using the estimation results from specification (4) in Table 1. To eliminate seasonal fluctuations, only weekday observations are used for the stay-at-home measure

**Fig. 4:**
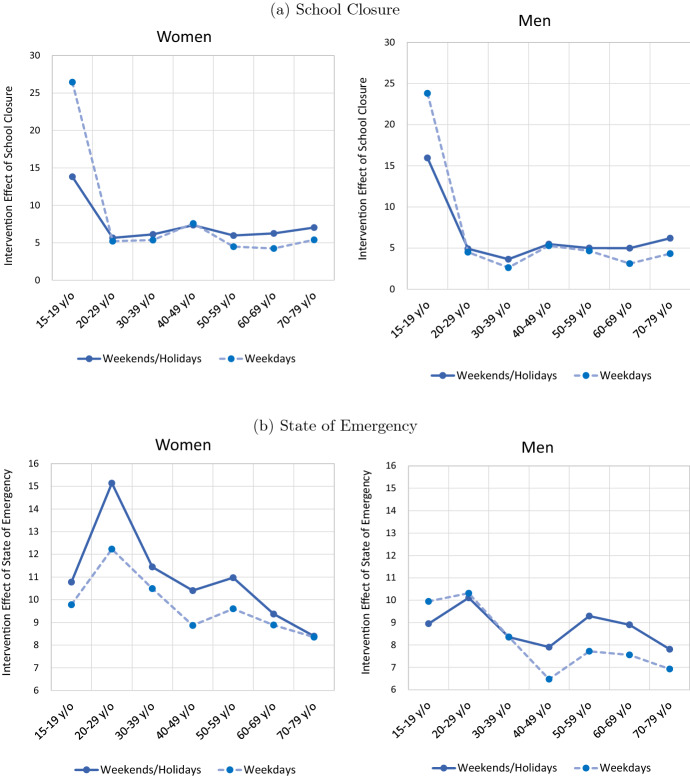
Intervention Effect by Gender, Age, and Weekday/Weekend. Note: The figures show the coefficient estimates presented in Tables 2 and 3

**Fig. 5:**
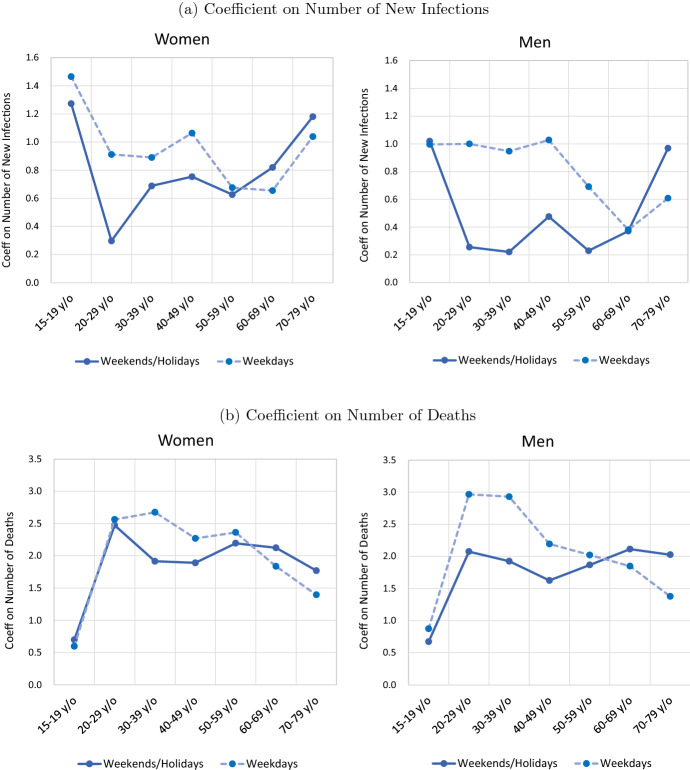
Information Effect by Gender, Age, and Weekday/Weekend. Note: The figures show the coefficient estimates presented in Tables 2 and 3

**Fig. 6:**
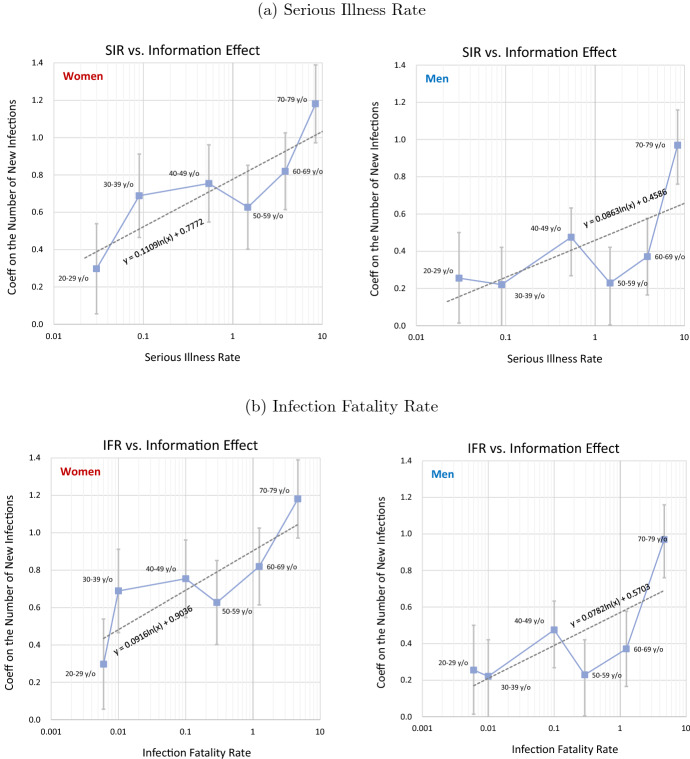
Serious Illness Rate, Infection Fatality Rate, and Information Effect. Note: The serious illness and infection fatality rates are the values for January to April 2020 taken from MHLW (2020). The coefficients for the number of new infections are taken from Tables 2 and 3. The vertical lines represent the error bar for each estimate, i.e., the estimated coefficient ± standard error

**Fig. 7:**
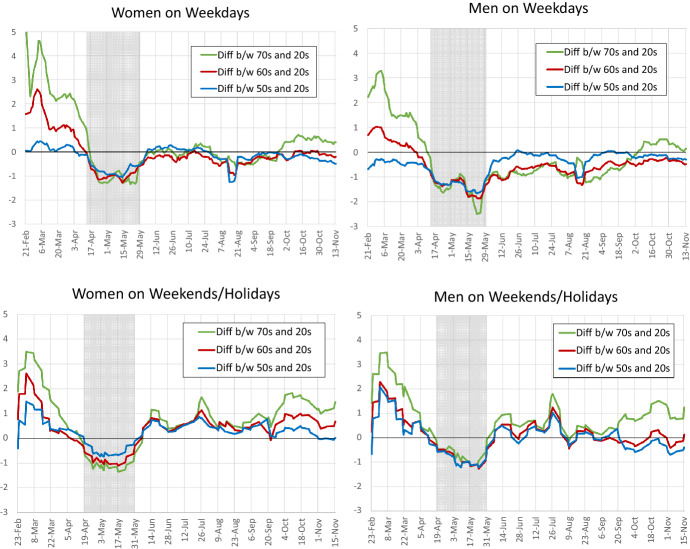
Differences of the Coefficients on the Number of New Infections Between Age Groups. Note: The shaded area represents the period of the state of emergency (April 7 to May 25, 2020)

**Fig. 8:**
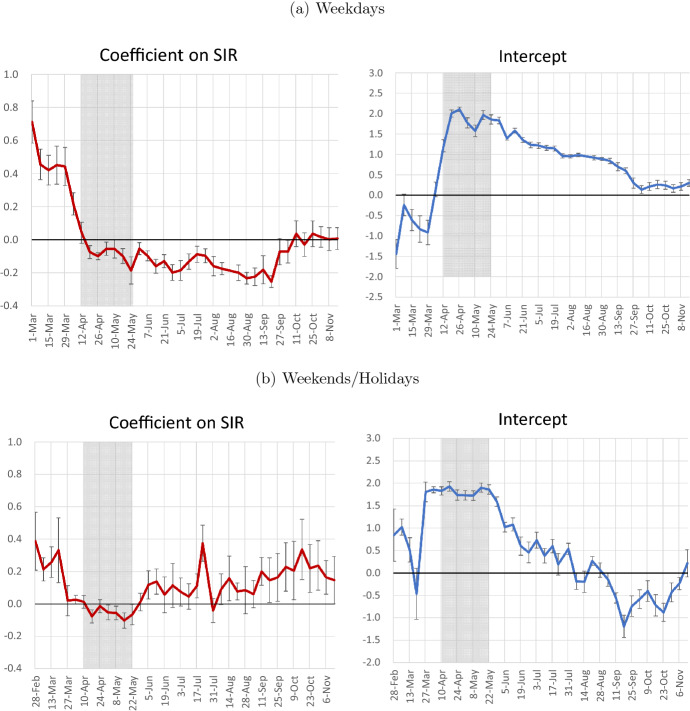
Second Stage Regression. Notes: The dependent variable is the arcsinh transform of the coefficient on the number of new infections by gender and age. Independent variables are the interaction terms of the arcsinh transform of the severe illness rate and the week dummies as well as a dummy for women and the week dummies. The red line represents the coefficient for the interaction terms of the age-specific severe illness rate and the week dummies, while the blue line shows the coefficient for the week dummies. The error bars for each week represent the range of the estimate$\pm 1.645\times$robust standard error. For the severe illness rate, the values from MHLW (2020) for January–April and June–August 2020 are used. For May 2020, the average of the values for January–April and June–August 2020 is used, while for the period from September onward, the value for June–August 2020 is used. The shaded area represents the period of the state of emergency (April 7–May 25, 2020)

The blue line in Fig. 1 is the stay-at-home measure created using mobile phone location data (details of how the measure is constructed are provided below). The line shows the extent to which Tokyo residents refrained from leaving their home compared to January 2020, before the pandemic. From March to May, the stay-at-home measure tended to increase as the number of new infections rose. However, during the second wave of infections from June to August, the stay-at-home measure hardly responded to the increase in the number of new infections. A similar lack of response can also be observed during the start of the third wave in November. In the first wave, people refrained from going out when they were exposed to information that the pandemic was worsening; however, after the first wave had subsided, people became less sensitive to information about the pandemic.

## Methodology

We estimate daily stay-at-home measures by age and gender for 47 prefectures to generate panel data. The observation period is from January 6 to November 15. We use the panel data for the following identification.

We start by identifying the intervention effect. Government policies such as the declaration of the state of emergency and school closures occurred at different times across prefectures, and by using these differences in timing, it is possible to determine whether the intervention or the information effect was responsible for changes in people’s behavior. For example, a state of emergency was declared in Tokyo on April 7, but at that time no state of emergency was declared for Tochigi prefecture, which is located 100 km north of Tokyo, about an hour away on the bullet train. A state of emergency was declared in Tochigi on April 16. Therefore, there was no intervention effect in Tochigi prefecture from April 7 to 15. However, people in Tochigi prefecture were aware that the state of emergency had been declared in Tokyo, so there was an information effect, and the stay-at-home measure rose accordingly. On the other hand, people in Tokyo refrained from leaving home due to both the intervention and information effects, and the stay-at-home measure rose. Therefore, assuming that residents in Tokyo and Tochigi had the same information about the pandemic and responded to it in the same way, we can extract the intervention effect by observing the difference in the stay-at-home measure between the two prefectures. It is important to note that even if the information on the pandemic is the same, the changes in behavior resulting from fear will differ between the old and the young. Therefore, it is crucial to measure the difference in the stay-at-home measure for the same age group in both prefectures, such as the old in Tokyo and the old in Tochigi Prefecture. As for the declaration of the state of emergency, not only did the time when it was declared differ across prefectures, but the lifting also occurred in three waves, so that differences in the timing of the lifting of the state of emergency can also be used to identify the intervention effect. Similarly, with regard to school closures, the timing of when school closures were lifted varies widely across prefectures, and this can also be used to identify the intervention effect. Meanwhile, since all measures against COVID-19 are carried out at the prefectural level, there are few differences across smaller administrative units within the same prefecture such as municipalities.

Thus, we use the variation in the stay-at-home measure across prefectures to identify the intervention effect. On the other hand, we use the variation in the stay-at-home measure across age groups to identify the fear effect. For example, suppose that the number of new infections in Tokyo spikes on a given day, and we want to extract the fear effect based on the change in the stay-at-home measure for Tokyo. Further, for simplicity, let us consider the extreme case in which the risk of serious illness and death among the young is zero, while it is high for the old. Because there is no risk for the young, they have no fear. Therefore, a rapid rise in new infections will not prevent them from going out. In contrast, when the old hear about a rise in new infections, they will be afraid and refrain from going out. Therefore, the fear effect can be extracted by subtracting the stay-at-home measure for the young from the stay-at-home measure for the old.

The empirical approach used in this study is as follows. Denote the stay-at-home measure at time *t* for age group *a* in prefecture *r* by $y_{art}$. Moreover, denote the number of new infections at time *t* in prefecture *r* by ${{\tilde{m}}}_{rt}$. The distribution of the number of new infections is skewed to the right because the number of new infections is much larger in a small number of prefectures such as Tokyo than most other prefectures. While many existing studies use logarithms to cope with such highly skewed distributions, for some of the prefectures in Japan the number of new infections is zero on some days, so that we cannot take logarithms. Following Goolsbee & Syverson (2021), we transform ${{\tilde{m}}}_{rt}$ using the inverse hyperbolic sine (or arcsinh) transformation. Specifically, we define $m_{rt}\equiv \ln ({{\tilde{m}}}_{rt}+\sqrt{{{\tilde{m}}}_{rt}^{2}+1})$. Similarly, we apply the inverse hyperbolic sine transformation to the number of deaths in each prefecture and denote this by $n_{rt}$. The estimation equation used in this study is as follows:

1 $$\begin{aligned}&y_{art}=\mu _{ar}+\underbrace{\alpha _{1a}D_{rt}(\text{ Emergency } \text{ declaration})+\alpha _{2a}D_{rt}(\text{ School } \text{ closure})} _{\text{ Intervention } \text{ effect }}\nonumber \\&\quad +\underbrace{\beta _{1a}m_{rt}+\beta _{2a}n_{rt}+\gamma _{at}}_{\text{ Information } \text{ effect }}+\epsilon _{art} \end{aligned}$$

where $\mu _{ar}$ represents the effect unique to the combination of age group *a* and prefecture *r*. $D_{rt}(\text{ Emergency } \text{ declaration})$ is a dummy variable that takes 1 when the state of emergency is active at time *t* in prefecture *r*, and 0 otherwise. Similarly, $D_{rt}(\text{ School } \text{ closure})$ is a dummy variable that takes 1 when schools are closed at time *t* in prefecture *r*, and 0 otherwise. Note that, when declaring the state of emergency, the Japanese government and local governments such as the Tokyo Metropolitan Government made the following requests: (1) households were asked to reduce their contacts with others by 80% by refraining from going out; (2) employers were asked to reduce the number of people coming in to work by 40% by introducing remote work; (3) theaters, meeting facilities, and entertainment establishments (nightclubs, live music venues, karaoke boxes, etc.) were requested to close, while restaurants were asked to refrain from operating after 8 pm. The dummy variable $D_{rt}(\text{ Emergency } \text{ declaration})$ captures the combined effect of these requests on the stay-at-home measure.^2^

In Eq. (1), the coefficients of the two dummy variables for intervention policies, $\alpha _1$ and $\alpha _2$, are assumed to be age dependent. Highlighting that hospitalization and fatality rates vary substantially between age groups, Acemoglu et al. (2020) show that targeted lockdowns focusing on the old, rather than uniform lockdowns for all age groups, would be more desirable in terms of effectively controlling infections while minimizing the impact on economic activity. If Japan’s intervention policies meet the criteria of optimal intervention policies proposed by Acemoglu et al. (2020), then $\alpha _{1}$ and $\alpha _{2}$ should be increasing functions of *a*.

Cochrane (2020) modifies the SIR model to incorporate that people reduce contacts in response to the severity of the disease by assuming that the transmission rate of the virus depends on the number of new infections and deaths. Following Cochrane (2020), the fourth and fifth terms on the right-hand side of Eq. (1) assume that people decide whether or not to go out in response to information about the pandemic such as the number of new infections and deaths. The important point here is that the two coefficients $\beta _{1}$ and $\beta _{2}$ in the fourth and fifth terms depend on age. This represents the fear effect. In other words, the risk of serious illness and death increases with age, and hence the level of fear and the sensitivity of the response to information on infections also increase with age.^3^ Specifically, $\beta _{1a}$ and $\beta _{2a}$ are assumed to be increasing functions of the serious illness rate $\theta _{a}$. Next, the sixth term on the right-hand side of Eq. (1) represents the response of people to information on the pandemic other than the number of new infections and deaths. The $\gamma _{at}$ in the sixth term is the time fixed effect and takes a value specific to age group *a*. We assume that people respond to the number of new infections and deaths in their own prefecture and that all other information on the pandemic is identical across prefectures.

Equation (1) is estimated in two steps. In the first step, (1) is estimated for each age group to obtain estimates of $\alpha _{1a}$, $\alpha _{2a}$, $\beta _{1a}$, and $\beta _{2a}$ for each age group *a*. In the second step, we use the variation across age groups in the estimates of $\beta _{1a}$ and $\beta _{2a}$ to estimate

2 $$\begin{aligned} \eta _{k}\equiv \frac{d \ln \beta _{ka}}{d \ln \theta _{a}} \qquad \quad k=1,2 \end{aligned}$$

Specifically, we transform $\beta _{1a}$ and $\theta _{a}$ using the arcsinh transformation and conduct a simple regression where the former is the dependent variable and the latter is the independent variable to obtain $\eta _{1}$. We do the same for $\eta _{2}$.

We would like to add the following remarks on the estimation using Eq. (1). First, while we assume that, except for information on new infections and deaths, residents in all prefectures are exposed to the same information on infections, in practice the information people have may differ across regions. In Sect. 6, we divide Japan into seven regions and use a specification that allows for different time dummies and thus a different $\gamma _{at}$ for each region. Second, it is possible that the parameter for the fear effect, $\beta _{ka}$, is not constant but may change over time. For instance, as people experience the first, second, and third waves of the pandemic, the response to changes in the number of new infections may weaken. In Sect. 6, we also conduct estimations allowing for changes in $\beta _{ka}$ over time. Finally, it is possible that people’s decisions to refrain from going out differs between weekdays and weekends/holidays. Whether or not people go out on weekdays depends on their employer’s decision, such as whether or not they introduce remote work. In Sect. 6, we also estimate $\alpha$ and $\beta$ allowing for differences between weekdays and weekends/holidays.

## Data

### The stay-at-home measure

For our location data, we use the “Mobile Spatial Statistics” provided by DoCoMo Insight Marketing.^4^ The Mobile Spatial Statistics provide location records of about 78 million DoCoMo mobile phones at 10-min intervals. Specifically, the mobile phone base stations in a particular area know which mobile phones are in the area. Based on this information, and dividing Japan into a mesh of 500m $\times$ 500m squares, DoCoMo compiles and publishes data on how many mobile phones are in a certain mesh element at a particular time (in 10-min intervals), together with information on the age and gender of the owners of those mobile phones as well as the municipality they live in. For our analysis, we use such information on mobile phone owners’ age and divide them into seven age groups (15–19, 20–29, 30–39, 40–49, 50–59, 60–69, and 70–79 years of age).

Using these data, we construct our stay-at-home measure by prefecture and age group in the following steps. The first step consists of the detection of residential areas. For a certain mesh element, we count the average number of people in the time from midnight to 5 am and take this as the nighttime population of that mesh element. Similarly, we count the number of people in the time from 9 am to 5 pm and take this as the daytime population of that mesh element. Based on this information, we identify an area as residential if the daytime population is smaller than the nighttime population multiplied by a parameter prespecified to be in the range from 0 to 1. We set the parameter to 0.8 but confirm that the results are essentially the same when we set the parameter to 0.7 or 0.9.

The second step is the calculation of the ratio of those leaving their homes. For mesh elements that in the first step were identified as residential areas, we calculate the number of people leaving home by counting the nighttime population and daytime population on a certain day and subtracting the daytime population from the nighttime population. Next, for each prefecture, we calculate the number of people leaving their homes each day by aggregating the number of people that have left their homes in each mesh element.

Finally, we take the number of persons leaving their homes in January 2020 (January 6 to 31), i.e., before the outbreak of COVID-19, as the number of persons leaving their homes during normal times, and then calculate for each prefecture and day the percentage difference from the number of people leaving their homes during normal times. We use the deviation rate multiplied by $-1$ as the stay-at-home measure. See Mizuno et al. (2021) for details of the calculation procedure for the stay-at-home measure.

Note that our stay-at-home measure is constructed based on cellular base station data rather than GPS data. If we were interested in how many people are in a particular commercial area, such as stores, stations, or parks, GPS data would provide useful and reliable information, since mobile apps requiring a GPS connection, such as Google Maps, are typically on in such commercial areas. However, GPS data may not be that useful when users are at home and therefore do not use location services frequently. In contrast, cellular base station data continues to provide reliable information on users’ location even when they are at home as mobile phones stay connected to the nearest cellular base station. Thus, cellular base station data is more suitable for our analysis, given that the focus of this study is the extent to which people choose to stay at home in response to the outbreak of the pandemic as well as governments’ interventions.

### Number of new infections

The central government and prefectures announce the number of new infections daily. The date of infection is the day when a doctor confirms that a person’s polymerase chain reaction (PCR) test was positive (test result date).^5^ We use figures from the database constructed and published by NHK (Nippon Hoso Kyokai, or Japan Broadcasting Corporation).^6^ The number of new infections varies greatly depending on the day of the week. In the analysis here, we assume that people make their decision on whether to leave their homes or not based on the trend in new infections over the preceding week, and we therefore use the moving average over the preceding week including the day in question.

### Number of deaths

We use the data published by the government and local governments and aggregated by prefecture by NHK. The number of deaths in Japan is low compared to the United States, Europe, and other countries (as of January 31, 2021, the total number of deaths in Japan was 5753). For this reason, 92% of all cells in the daily data by prefecture are zero. For the number of deaths, we use the moving average over the preceding week including the day in question, since it is likely that people make decisions on whether to go out or not by paying attention to trends rather than to daily figures. The number of cells containing zero when we use the 7-day moving average is 79% of the total.

### Serious illness rate and fatality rate

The severe illness rate is the sum of the number of severe cases and deaths divided by the number of infected persons, including asymptomatic cases. The fatality rate is the number of deaths divided by the number of infected persons, including asymptomatic cases. The original data are surveillance data gathered by the Ministry of Health, Labour and Welfare. Both the severe illness rate and the fatality rate are based on confirmed cases from January to April 2020 and on confirmed cases from June to August 2020. The age groups are: under 10 years of age, 10–19, 20–29, $\cdots$, 80–89  years of age, and 90 years and older. See MHLW (2020) for details.

### Government measures against the spread of COVID-19

School closures On February 27, the government requested all elementary schools, junior high schools, high schools, and special needs schools to be closed from March 2 onwards. In response to this, all prefectures except Hokkaido closed schools from March 2.^7^ We construct a dummy variable for school closures, *School closure*, that takes 1 during the period schools were closed in a particular prefecture, and 0 otherwise. Specifically, except for Hokkaido, the dummy takes 1 from March 2, the day that schools were closed, until the date on which schools were opened again in a particular prefecture. For Hokkaido, the dummy takes a value of 1 from February 27, the day on which schools were closed in that prefecture. The date of the reopening of schools varies widely across prefectures: the earliest date was April 6, while the latest date was June 1. Note that if a prefecture closed schools again within a short time of reopening them, we do not regard this as reopening.

State of emergency The government declared a state of emergency for seven prefectures (Saitama, Chiba, Tokyo, Kanagawa, Osaka, Hyogo, Fukuoka) on April 7, and expanded the state of emergency to all prefectures on April 16.^8^ The state of emergency was lifted in 39 prefectures with few infections on May 14, in three more prefectures on May 21, and finally in the remaining five prefectures including Tokyo on May 25. We also construct a dummy variable for the state of emergency, *State of Emergency*, that takes 1 when the state of emergency is active in a particular prefecture, and 0 otherwise. For example, for Tokyo, the *State of Emergency* dummy is set to 1 from April 8, the day after a state of emergency was declared for Tokyo, to May 25, when it was lifted.

### Other factors affecting whether people left their homes

To take other factors into account, a rain dummy (*Rain*) is used. The rain dummy takes 1 if the amount of precipitation in the prefectural capital was greater than 0, and takes 0 otherwise. Precipitation data were obtained from the Japan Meteorological Agency website.

## Results

### Baseline regressions

Let us begin by examining how the results we obtained in our earlier study (Watanabe & Yabu, 2020) change when we extend the estimation period. Specification (1) in Table 1 is exactly the same as that used in Watanabe & Yabu, (2020), and the main explanatory variables are the dummy variables for the two intervention policies, i.e., the state of emergency declaration and closure of schools, and the number of new infections to capture the information effect. As fixed effects, we include prefecture dummies and time dummies for each of the seven regions. To take into account that the number of mobile phone owners differs across prefectures, the estimation is conducted employing weighted least squares using the number of mobile phone owners as weights.

**Table 1:** Baseline Regressions

	(1)	(2)	(3)	(4)
School closure	6.454***	6.021***	6.111***	5.054***
	(1.031)	(0.988)	(1.023)	(1.192)
State of emergency	9.602***	9.067***	8.983***	5.363***
	(1.152)	(0.979)	(0.970)	(1.208)
No. of new infections	0.810**		0.496**	Time-varying
	(0.306)		(0.223)	
No. of deaths		2.326***	1.923***	Time-varying
		(0.721)	(0.634)	
Rain	0.661***	0.709***	0.690***	0.688***
	(0.199)	(0.200)	(0.198)	(0.209)
Obs.	14805	14805	14805	14805
Adjusted $R^2$	0.957	0.958	0.958	0.974
FEs	Prefecture	Prefecture	Prefecture	Prefecture
	Day$\times$Region	Day$\times$Region	Day$\times$Region	Day$\times$Region

Figures in parentheses are cluster-robust standard errors. *, **, and *** denote statistical significance at the 10%, 5% and 1% level, respectively. For the number of new infections within prefectures, the inverse hyperbolic sine transforms ($\text{ arcsinh }(x)=\ln (x+\sqrt{x^{2}+1})$) were used. Similarly, the number of deaths were transformed using the inverse hyperbolic sine transformation. The coefficients for the number of new infections and the number of deaths are shown in Fig. 2

**Table 2:** Intervention and Information Effects by Gender and Age: Weekdays

Men	15–19 y/o	20–29 y/o	30–39 y/o	40–49 y/o	50–59 y/o	60–69 y/o	70–79 y/o
School closure	23.797***	4.500***	2.619***	5.282***	4.650***	3.112***	4.317***
	(4.287)	(1.242)	(1.062)	(1.073)	(1.012)	(0.817)	(1.065)
State of emergency	9.947***	10.313***	8.359***	6.472***	7.719***	7.556***	6.928***
	(2.104)	(1.568)	(1.198)	(0.836)	(1.067)	(1.005)	(0.917)
No. of new infections	0.996***	1.000***	0.947***	1.028***	0.692***	0.380***	0.608***
	(0.387)	(0.271)	(0.337)	(0.307)	(0.260)	(0.188)	(0.144)
No. of deaths	0.873	2.965***	2.928***	2.193***	2.021***	1.847***	1.377***
	(0.681)	(0.920)	(0.866)	(0.657)	(0.598)	(0.553)	(0.425)
Rain	0.289	0.351*	0.403***	0.477***	0.531***	0.960***	2.251***
	(0.359)	(0.192)	(0.145)	(0.151)	(0.167)	(0.229)	(0.450)
Obs.	9917	9917	9917	9917	9917	9917	9917
Adjusted $R^2$	0.951	0.934	0.925	0.937	0.943	0.933	0.932
FEs	Prefecture	Prefecture	Prefecture	Prefecture	Prefecture	Prefecture	Prefecture
	Day$\times$Region	Day$\times$Region	Day$\times$Region	Day$\times$Region	Day$\times$Region	Day$\times$Region	Day$\times$Region

Women	15–19 y/o	20–29 y/o	30–39 y/o	40–49 y/o	50–59 y/o	60–69 y/o	70–79 y/o
School closure	26.453***	5.199***	5.354***	7.575***	4.473***	4.233***	5.390***
	(4.506)	(1.304)	(1.277)	(1.452)	(1.128)	(0.942)	(1.205)
State of emergency	9.782***	12.230***	10.491***	8.866***	9.601***	8.884***	8.352***
	(2.027)	(1.523)	(1.293)	(0.930)	(1.218)	(1.020)	(0.959)
No. of new infections	1.466***	0.912***	0.891***	1.064***	0.676***	0.655***	1.039***
	(0.398)	(0.233)	(0.296)	(0.310)	(0.218)	(0.132)	(0.148)
No. of deaths	0.598	2.563***	2.674***	2.270***	2.363***	1.837***	1.395***
	(0.528)	(0.833)	(0.749)	(0.636)	(0.676)	(0.577)	(0.426)
Rain	0.240	0.375*	0.535***	0.433***	0.571***	1.080***	2.060***
	(0.369)	(0.198)	(0.179)	(0.177)	(0.184)	(0.251)	(0.411)
Obs.	9917	9917	9917	9917	9917	9917	9917
Adjusted $R^2$	0.955	0.949	0.946	0.951	0.950	0.946	0.951
FEs	Prefecture	Prefecture	Prefecture	Prefecture	Prefecture	Prefecture	Prefecture
	Day$\times$Region	Day$\times$Region	Day$\times$Region	Day$\times$Region	Day$\times$Region	Day$\times$Region	Day$\times$Region

Figures in parentheses are cluster-robust standard errors. *, **, and *** denote statistical significance at the 10%, 5% and 1% level, respectively. For the number of new infections within prefectures, the inverse hyperbolic sine transforms ($\text{ arcsinh }(x)=\ln (x+\sqrt{x^{2}+1})$) were used. Similarly, the number of deaths were transformed using the inverse hyperbolic sine transformation

The results for specification (1) show that the coefficient on the school closure dummy is 6.5, indicating that school closures had the effect of raising the stay-at-home measure by 6.5 percentage points. Meanwhile, the state of emergency declaration had the effect of raising the stay-at-home measure by 9.6 percentage points. The corresponding coefficient estimates in Watanabe & Yabu (2020) using data for the first wave only were 4.8 and 7.0, respectively. Thus, both coefficients in our new estimate are slightly larger than in the previous estimate. Next, the coefficient for the number of new infections is 0.8, meaning that a 1% increase in the number of new infections in a prefecture raised the stay-at-home measure for that prefecture by 0.008 percentage points. This is considerably smaller than the coefficient estimate of 2.2 obtained in Watanabe & Yabu (2020). This result confirms the observation from Fig. 1 that since June 2020 the stay-at-home measure has become less responsive to changes in the number of new infections.

Many studies on the United States, Europe, China, and other countries use the number of deaths as the variable to represent the spread of infections. The reason is that the number of new infections is regarded to represent an inaccurate picture of the true situation since it is affected by changes in the number of tests. Therefore, in specification (2) we use the number of deaths instead of the number of new infections to represent the information effect. The coefficient on the number of deaths is 2.3 and is statistically significant, indicating that a 1% increase in the number of deaths in a given prefecture is associated with a 0.023 percentage point increase in the stay-at-home measure of that prefecture. Further, in specification (3), we include both the number of new infections and the number of deaths in the estimation and find that the coefficients for both are statistically significant. The number of deaths, although small, had a non-negligible impact on the stay-at-home measure.

Next, in specification (4) we use the interaction term of the number of new infections and time dummies and the interaction term of the number of deaths and time dummies as explanatory variables to examine how the coefficients on the number of new infections and deaths changed over time. Figure 2 shows the coefficient estimates. The coefficient for the number of new infections began to rise at the end of March and reached a value of 3 in April during the state of emergency. It began to decline when the state of emergency was lifted and continued to decline until it reached almost zero in September, where it subsequently more or less remained.^9^ This shows that the pattern seen in Fig. 1 for Tokyo, where despite the increase in the number of new infections during the second and third waves, the stay-at-home measure did not increase, can also be observed for Japan as a whole. Next, turning to the coefficient for the number of deaths, this shows a large jump at the beginning of March, but since then it has remained around 2. However, in autumn, like the coefficient for the number of new infections, it dropped to almost zero. A possible reason for the decline in the coefficients for the number of new infections and deaths may be that the fear of infection has been decreasing. This point will be examined in more detail in Sect. 6.4.

Figure 3 shows the results of decomposing changes in the stay-at-home measure for Tokyo into the intervention effect and the information effect using the estimation results of specification (4). The intervention effect shows how much the two intervention policies, i.e., school closures and the emergency declaration, contributed to the increase in the stay-at-home measure. The information effect shows how much information on the number of new infections and deaths contributed to the increase in the stay-at-home measure by prompting people to refrain from going out. Moreover, the response of people to other information about the pandemic can be considered to be captured by the time fixed effect, and we include this in the information effect. As can be seen from the figure, the contribution of the two interventions is limited. On the other hand, the contribution of the information effect is large. This is identical to the result in our previous study (Watanabe & Yabu 2020). However, here we find that the contribution of the information effect became smaller from the second half of September. Particularly noteworthy is that the contribution of the information effect linked to the number of new infections has become quite small. Meanwhile, the number of new infections itself has not decreased in Tokyo since the latter half of September—in fact, it has increased. On the other hand, as seen in Fig. 2, people’s response to the change in the number of new infections has weakened, and as a result, the contribution of the information effect through the number of new infections has decreased.

### Intervention effect by gender, age, and weekday/weekend

Next, we examine the intervention effect by gender, age, and weekday/weekend. In Tables 2 and 3, we use specification (3) from Table 1 to conduct estimates by gender and age. Table 2 shows the results of the same estimation using only weekday data, while Table 3 shows the results using only weekend/public holiday data. With a few exceptions, all the estimated coefficients are statistically significant.

**Table 3:** Intervention and Information Effects by Gender and Age: Weekends/Holidays

Men	15–19 y/o	20–29 y/o	30–39 y/o	40–49 y/o	50–59 y/o	60–69 y/o	70–79 y/o
School closure	15.946***	4.908***	3.639***	5.474***	4.994***	4.988***	6.195***
	(2.958)	(1.189)	(1.005)	(1.251)	(1.147)	(1.288)	(1.710)
State of emergency	8.952***	10.109***	8.351***	7.904***	9.294***	8.902***	7.810***
	(1.262)	(0.881)	(0.771)	(0.758)	(1.016)	(0.892)	(0.861)
No. of new infections	1.020***	0.255	0.221	0.475***	0.229	0.371*	0.969***
	(0.267)	(0.245)	(0.200)	(0.157)	(0.192)	(0.206)	(0.190)
No. of deaths	0.671	2.075***	1.924***	1.625***	1.867***	2.113***	2.026***
	(0.618)	(0.717)	(0.577)	(0.620)	(0.672)	(0.610)	(0.547)
Rain	1.792***	1.473***	1.648***	1.659***	1.797***	2.053***	3.438***
	(0.523)	(0.418)	(0.359)	(0.371)	(0.423)	(0.500)	(0.763)
Obs.	4888	4888	4888	4888	4888	4888	4888
Adjusted $R^2$	0.947	0.927	0.938	0.957	0.953	0.951	0.950
FEs	Prefecture	Prefecture	Prefecture	Prefecture	Prefecture	Prefecture	Prefecture
	Day$\times$Region	Day$\times$Region	Day$\times$Region	Day$\times$Region	Day$\times$Region	Day$\times$Region	Day$\times$Region

Women	15–19 y/o	20–29 y/o	30–39 y/o	40–49 y/o	50–59 y/o	60–69 y/o	70–79 y/o
School closure	13.816***	5.645***	6.113***	7.350***	5.962***	6.244***	7.026***
	(2.244)	(1.172)	(1.273)	(1.376)	(1.146)	(1.316)	(1.764)
State of emergency	10.776***	15.138***	11.444***	10.405***	10.972***	9.372***	8.394***
	(0.882)	(1.227)	(1.063)	(0.998)	(1.200)	(1.012)	(0.885)
No. of new infections	1.274***	0.297	0.689***	0.754***	0.627***	0.820***	1.181***
	(0.282)	(0.241)	(0.223)	(0.207)	(0.225)	(0.206)	(0.209)
No. of deaths	0.699	2.474***	1.915***	1.891***	2.195***	2.124***	1.770***
	(0.525)	(0.762)	(0.623)	(0.637)	(0.704)	(0.606)	(0.477)
Rain	0.954**	0.862**	1.590***	1.425***	1.267***	1.752***	2.880***
	(0.418)	(0.325)	(0.363)	(0.347)	(0.359)	(0.443)	(0.648)
Obs.	4888	4888	4888	4888	4888	4888	4888
Adjusted $R^2$	0.959	0.957	0.965	0.969	0.962	0.962	0.962
FEs	Prefecture	Prefecture	Prefecture	Prefecture	Prefecture	Prefecture	Prefecture
	Day$\times$Region	Day$\times$Region	Day$\times$Region	Day$\times$Region	Day$\times$Region	Day$\times$Region	Day$\times$Region

Figures in parentheses are cluster-robust standard errors. *, **, and *** denote statistical significance at the 10%, 5% and 1% level, respectively. For the number of new infections within prefectures, the inverse hyperbolic sine transforms ($\text{ arcsinh }(x)=\ln (x+\sqrt{x^{2}+1})$) were used. Similarly, the number of deaths were transformed using the inverse hyperbolic sine transformation

Based on the results in Tables 2 and 3, Fig. 4 looks at how the intervention effect depends on gender and age. Starting with the impact of school closures on the stay-at-home measure for weekdays, we find that school closures had a large intervention effect for 15–19 year olds (i.e., those of school age), while the intervention effect for the other age groups, although not zero, was small. In terms of gender, restraint from going out was slightly larger among women, suggesting that the burden of housework and childcare during school closures may have disproportionately fallen on women. Further, what is interesting about the estimation results for the intervention effect of school closures is that a statistically significant intervention effect is observed even for weekends and public holidays. This suggests that the school closures may have reduced interaction between students and parents at school, leading to a reduction in weekend activities.

Turning to the intervention effect of the state of emergency declaration, the results show that this is dependent on age: with the exception of the 15–19 age group, the intervention effects get weaker with age. The difference between younger and older age groups is particularly pronounced for women on weekends/holidays and is statistically significant when comparing women in their 20s with those in their 60s and 70s. Thus, while Acemoglu et al. (2020) argue that optimal intervention policies would impose restrictions on those most at risk, namely, older age groups, Japan’s emergency declaration had the opposite effect in that the extent to which people refrained from going out was more pronounced among the young.

When declaring the state of emergency, the Japanese government and local governments such as the Tokyo Metropolitan Government asked households to reduce their contacts with others by 80% by refraining from going out, while employers were asked to reduce the number of people coming in to work by 40% by introducing remote work. Further, theaters, meeting facilities, and entertainment establishments (nightclubs, live music venues, karaoke boxes, etc.) were requested to close, while restaurants were asked to refrain from operating after 8 pm. The request to employers to curtail the number of workers required to come in to work had the effect of reducing outings by those of working-age, while the request for places such as entertainment establishments and restaurants to close or refrain from operating after 8pm had the effect of discouraging those in their 20s to 50s—the main users of such places—from going out. Although the government did not intend to reduce outings of older age groups at higher risk of illness and death to a lesser extent than that of younger age groups, this ultimately was the effect of the state of emergency. This may have unnecessarily increased the number of people falling seriously ill, putting medical resources under strain and increasing the number of deaths. However, our finding that government intervention inadvertently may have had the opposite of the intended effect is not unique to Japan: as mentioned in Sect. 2, studies by Caselli et al. (2020) and Andersen et al. (2020), among others, using data for Europe, also show that government policies in these countries have restricted activities of the young to a greater extent than those of the old, suggesting that government intervention policies to prevent infections did not meet the criteria of optimal intervention policies proposed by Acemoglu et al. (2020).

Comparing the intervention effect of the emergency declaration between men and women of the same age group, we find that in all age groups women refrained from going out to a greater degree than men, both on weekdays and on weekends/holidays. This result is similar to the effect of school closures mentioned earlier. One possible explanation is that, as shown by Kikuchi et al. (2021) using data for Japan, women are more likely to be employed in contact-intensive sectors. Similarly, Caselli et al. (2020) found that in Italy, Spain, and Portugal, the decline in mobility during lockdowns was larger for women than for men. They suggest that this was because the burden of housework and looking after children during the lockdowns disproportionately fell on women, and because women are more likely to be employed in contact-intensive sectors and are therefore affected to a greater degree by the impact of lockdowns on employment. The results thus show that in major countries including Japan, government intervention policies have forced women to stay at home to a greater extent than men.

### Information effect by gender, age, and weekday/weekend

We now turn to the information—or fear—effect. Specifically, in Fig. 5, we compare the information effect by age, gender, and weekday/weekend using the results from Tables 2 and 3. The upper panel shows the coefficients for the number of new infections, while the lower panel shows the coefficients for the number of deaths. Starting with the coefficients for the number of new infections for women, we find that for weekends/holidays the coefficient tends to increase with age from those in their 20s. For example, the coefficients for women in their 20s (0.3), 60s (0.8), and 70s (1.2) are very different, and some of these differences are statistically significant. This finding suggests that the older the age group, the greater is the fear of infection in response to news about an increase in the number of new infections, and the greater is the restraint from going out. On the other hand, for weekdays, the coefficient tends to slightly decrease with age from those in their 20s to those in their 50s and 60s. However, the coefficient for those in their 70s then increases substantially from those in their 50s and 60s. The pattern that the coefficient increases with age for weekends/holidays but, conversely, decreases with age for weekdays is also observed for men. The reason why the way that the information effect depends on age differs between weekdays and weekends/holidays likely is that whereas on weekends and holidays it is individuals themselves who decide whether to stay at home in response to news about the pandemic, on weekdays this is largely up to employers. That is, those in their 20s to 50s tend to be active workers, and how they respond to an increase in the number of new infections largely depends on the decision of employers, such as whether they introduce remote working. On the other hand, older individuals that are already retired can decide for themselves how to respond to changes in the number of new infections.^10^

Next, looking at the coefficients on the number of deaths, both for men and women, the values for weekends/holidays are more or less identical across age groups for those in their 20s and upward. Thus, we do not find the same pattern as for the coefficient on the number of new infections, which increases with age. For weekdays, the coefficient tends to decrease with age for those in their 20s and upward. Cochrane (2020) argues that although the number of new infections and the number of deaths both provide information about the pandemic, the meaning for recipients differs substantially. If this is indeed the case, this implies that information about an increase in the number of new infections in a particular prefecture on a given day means that the risk of infection in that prefecture at that time has increased, and people react to that information by refraining from going out. In making their decisions, people will also take into account that the risk of serious illness and death varies with age. In contrast, news of an increase in the number of deaths is merely the result of an increase in the number of infections in the past and does not necessarily mean that the risk of infection on that day is particularly high. Of course, news of an increase in the number of deaths likely raises people’s fear and discourages them from going out. In fact, the coefficient on the number of deaths is positive for all age groups and statistically significant, except for the 15–19 age group. However, since this type of fear does not stem from the increased risk of infection on that day, it is not necessarily the case that older people, who are at higher risk of severe illness and death, are more afraid. The finding that the coefficient for weekends/holidays is almost identical across age groups can be interpreted as reflecting these circumstances.

Figure 6 shows how the coefficient on the number of new infections for each age group is related to the serious illness rate. The panel on the left is for women, while that on the right is for men, and, using the estimate for weekends/holidays for each age group from those in their 20s to those in their 70s, we look at the relationship between the coefficient on the number of new infections and the corresponding severe illness rate. As we saw in Fig. 5, whether people leave home on weekdays largely reflects the decisions of employers, so that we use the results for weekends/holidays here. Moreover, we exclude the age group of 15–19 year olds because whether they go out primarily reflects their parents’ decision. The severe illness rate is estimated based on the values for the period from June to August 2020. The severe illness rate on the horizontal axis is shown in logarithmic scale. Both for women and men, a positive correlation between the logarithm of the severe illness rate and the coefficient on the number of new infections can be observed. Running a simple regression, we find that a ten-fold increase in the severe illness rate is associated with an increase in the coefficient on the number of new infections of 0.25 for women and 0.20 for men. We will examine this in more detail in the next subsection.

### Changes in the information effect over time

As seen in Fig. 2, the coefficients on the number of new infections and deaths change substantially over time, indicating that the information effect is not constant over time. In this subsection, we therefore examine the coefficients on the number of new infections by gender and age allowing for changes in the coefficient over time. Specifically, we estimate Eq. (1) regarding $\beta _{1}$ as a time-varying parameter. However, we continue to estimate the coefficient on the number of deaths, $\beta _{2}$, as a fixed parameter because the prefecture-level number of deaths is zero on many days. The estimates of $\beta _{1}$ obtained in this manner are shown in Appendix Figs. 9 (for weekdays) and 10 (for weekends/holidays).

Using these estimates, Fig. 7 shows the differences in the coefficients on the number of new infections between different age groups. Specifically, the three lines represent the difference between the coefficients for people in their 70s and those in their 20s, the difference between the coefficients for people in their 60s and those in their 20s, and the difference between the coefficients for people in their 50s and those in their 20s. The upper panels show the results for weekdays, while the lower panels show the results for weekends/holidays.

The figure suggests the following. First, in the period from the end of February to the beginning of April, before the declaration of the state of emergency, the difference between those in their 70s and those in their 20s was large and positive. Similarly, the difference between those in their 60s and 20s was also positive. This pattern can be observed for both weekdays and weekends/holidays and for both women and men. On the other hand, while the difference between those in their 50s and those in their 20s was positive on weekends/holidays, it was zero or slightly negative on weekdays. The fact that the coefficients for the older age groups were higher than for those in their 20s suggests that people’s response to information about the pandemic reflected their level of risk of serious illness and death.

**Fig. 9:**
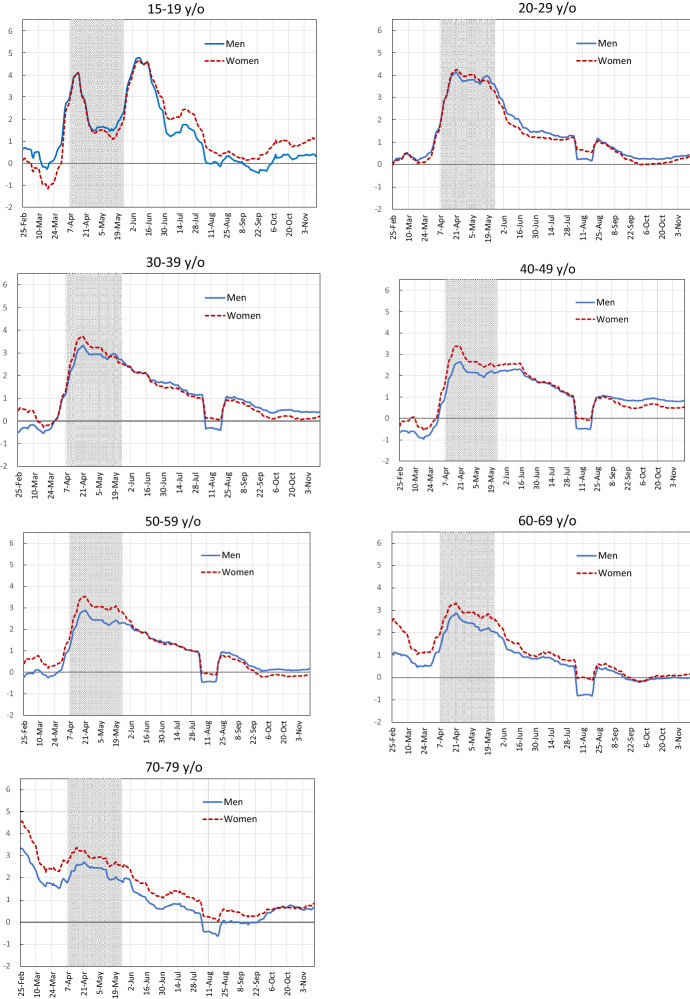
Coefficients on the Number of New Infections by Gender and Age: Weekdays. Notes: Estimated based on eq. (1) with the coefficient on the number of new infections, $\beta _{1}$, regarded as time varying while the coefficient on the number of deaths, $\beta _{2}$, as time invariant. The rain dummy is used. Only weekday observations are used. The shaded area represents the period of the state of emergency (April 7 to May 25, 2020)

**Fig. 10:**
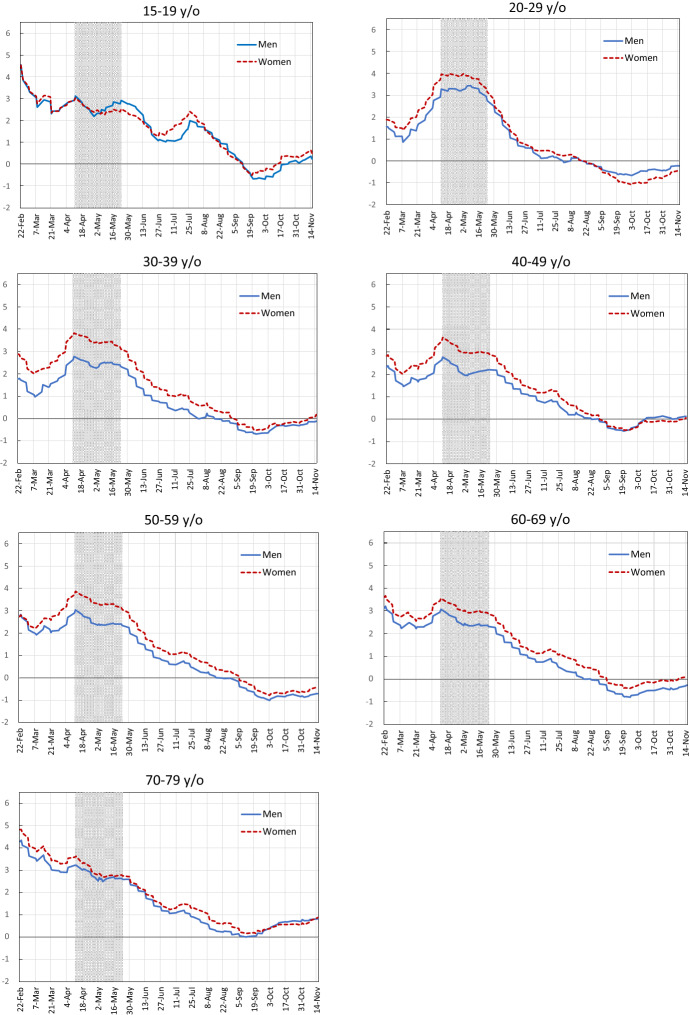
Coefficients on the Number of New Infections by Gender and Age: Weekends/Holidays. Notes: Estimated based on Eq. (1) with the coefficient on the number of new infections, $\beta _{1}$, regarded as time-varying, while the coefficient on the number of deaths, $\beta _{2}$, is regarded as time-invariant. The rain dummy is used. Only weekend/holiday observations are used. The shaded area represents the period of the state of emergency (April 7 to May 25, 2020)

Second, looking at the period of the state of emergency from April to May, the difference between the older age groups and those in their 20s turned negative. Taking a closer look at Figs. 9 and 10 shows why this sign reversal occurs: the coefficient for those in their 70s during the state of emergency fell from the level before the state of emergency. Moreover, while the coefficients for those in their 60s and 50s increased slightly compared to before the state of emergency, the increase was negligible. In contrast, the coefficients for people in their 20s increased markedly with the declaration of the state of emergency. A possible reason for the difference between the older age groups and those in their 20s is that those in their 20s may have been less aware of the pandemic at first, but this changed as the state of emergency was declared.^11^

Third, after the state of emergency was lifted in June, the difference between the older age groups and those in their 20s was negative or zero on weekdays as before the state of emergency, but it returned to a positive level for weekends/holidays. The fact that the difference returned to a positive level on weekends/holidays, when employers hold little sway over the decision whether people leave home, suggests that people’s response to information about the pandemic was commensurate with their risk of serious illness and death. Note that the estimates for each age group on weekends/holidays shown in Fig. 10 indicate that the coefficients for all age groups declined gradually after the state of emergency, before starting to slightly pick up again early October. During this process, the coefficients for the older age groups showed first a slower decline and then a quicker increase.

Next, Fig. 8 shows the results of estimating $\eta _{1}$ in Eq. (2) in order to examine the relationship between the coefficient on the number of new infections and the risk of severe illness. Specifically, we take the arcsinh transform of the coefficient on the number of new infections by gender and age as the dependent variable and regress this on the interaction term of the arcsinh transform of the severe illness rate by age and week dummies as well as a dummy for women and the week dummies. For the severe illness rate, we use the values for January–April and June–August 2020 as estimated by MHLW (2020). The red line in the figure depicts the coefficient on the severe illness rate multiplied by the week dummy and is an estimate of $\eta _{1}$. The blue line shows the coefficient on the week dummy. The error bars for each estimate represent the range of the estimate ±1.645 times the robust standard error.

As shown in Fig. 2, the coefficient on the number of new infections has been declining since June. If this downward trend is due to the weakening of the fear effect in Eq. (1), then $\eta _{1}$ (i.e., the red line in Fig. 8) should decrease over time and approach zero. However, looking at developments in $\eta _{1}$ after the state of emergency, we find that for weekdays, as shown in Fig. 8a, it stayed negative from June to September and then rose to close to zero in October. On the other hand, Fig. 8b for weekends/holidays indicates that the value was zero at the beginning of June but has been positive since then and for quite a few weeks is statistically significantly different from zero. These results (especially those for weekends/holidays) mean that the interpretation that $\eta _{1}$—and hence the fear effect—decreased after the state of emergency was lifted, and that this is the reason why the coefficient on the number of new infections decreased, is not appropriate.

On the other hand, looking at developments in the constant term represented by the blue line, we find that it increased around the time the state of emergency was declared and then gradually decreased once the state of emergency was lifted. Moreover, this pattern can be observed for both weekdays and weekends/holidays. A potential reason for this may be that factors other than fear, such as altruism, that initially led to changes in behavior may have gradually weakened during this period.^12^ Another possibility is that people’s perception of the severe illness rate deviated from the actual severe illness rate as estimated by MHLW (2020). Specifically, a possible explanation of the change in the constant term would be that the perceived severe illness rate for each age group uniformly increased with the declaration of the state of emergency and then uniformly decreased with the lifting of the state of emergency. Note that the estimates of $\eta _{1}$ in Fig. 8 are negative during the state of emergency. This also suggests that the value of $\theta$ used in the regression in this study may have deviated from the perceived severe illness rate during this period.

## Summary and policy implications

In this study, we examined how people’s behavior changed during the COVID-19 pandemic in terms of refraining from going out. As channels of such changes in behavior, we focused on the intervention effect (changes in people’s behavior as a result of government orders or requests) and the information effect (voluntary changes in behavior in response to information about the pandemic). Using smartphone location data, we created a stay-at-home measure by age group and gender to examine how the intervention and information effects depend on age.^13^

The main findings of this study are as follows. First, the age profile of the intervention effect of the emergency declarations issued in April and May 2020 shows that the degree to which older age groups with a higher risk of serious illness and death refrained from leaving home was smaller than that for younger age groups. This result indicates that the emergency declaration had the exact opposite effect of the targeted lockdowns proposed by Acemoglu et al. (2020), which aim to quarantine the old while allowing the young to carry on with economic activities, and may not have had the intended impact in terms of effectively minimizing both the public health consequences and economic losses. Underlying this, changes on the supply side of face-to-face services may have played an important role. That is, in the prefectures covered by the state of emergency, commercial facilities in shopping areas such as restaurants, theaters, and retail stores were requested to shorten their business hours or close, but the main users of such commercial facilities in shopping areas are those in their 20s to 50s. Thus, compared with those in the same age group in prefectures not affected by the state of emergency, those in the affected prefectures had fewer opportunities to use such commercial facilities and for this reason likely reduced their outings. In contrast, older age groups in their 60s and above were less likely to use such commercial facilities in the first place and thus were less affected by the supply-side effects of the emergency declaration.

Second, the age profile of the information effect shows that, unlike the intervention effect, the degree to which people stayed at home tended to increase with age for weekends and public holidays. This result suggests that people update their information about the pandemic daily and make decisions to refrain from going out based on their own risk of serious illness and death. In other words, what achieved a “targeted lockdown” à la Acemoglu et al. (2020) was not government interventions such as the declaration of a state of emergency but voluntary changes in people’s behavior. However, for weekdays, we observe no tendency for the degree to which people stayed at home to increase with age. On weekends and public holidays, people make decisions about whether or not to go out as consumers, and differences in the risk of serious illness and death across age groups tend to be reflected in differences in refraining from going out. On the other hand, on weekdays, people are both consumers and workers, and their decision whether or not to go out as workers depends largely on the decision of their employer (e.g., to shorten working hours or introduce working from home). This likely explains why the link between age and the degree to which people stay at home is relatively weak for weekdays.

Third, the information effect has been on a downward trend since the summer of 2020. While this trend applies to all age groups, it is relatively more pronounced among the young, so that the age profile of the information effect remains upward sloping, suggesting that people’s response to information about the pandemic is commensurate with their risk of serious illness and death.

Next, let us consider the policy implications of our findings. The state of emergency in April-May 2020 included measures to curb the activities of contact-intensive service industries, such as closing or reducing the opening hours of commercial facilities in shopping areas. Similar measures have been taken in the second state of emergency declared on January 7, 2021, which at the time of writing is still in effect in Tokyo and other prefectures. Our results suggest these supply-side measures have the effect of reducing activities of younger age groups to a greater extent than older age groups. This means that while their negative impact on economic activity is substantial, their effectiveness in terms of reining in the pandemic is limited. In fact, both during last year’s state of emergency and during the current state of emergency, the number of seriously ill elderly COVID patients increased rapidly, resulting in a severe shortage of hospital beds and medical resources. While the analysis in this study has focused only on the first state of emergency, it is important to examine whether the same age profile in terms of the effect—i.e., that it reduced outings by the young to a greater extent than the old—can be observed again for the second state of emergency. Moreover, based on the experience gained from the first and second state of emergency, a key point that warrants discussion is how the current type of government interventions could or should be modified to achieve optimal results in terms of effectively controlling infections while minimizing the adverse impact on economic activity.

Another issue is whether the government’s measures against the pandemic should be legally binding. Japan’s historical experience with government measures to control epidemics and other emergency measures means that the government has been reluctant to introduce legally binding measures.^14^ However, on February 3, 2021, Japan’s parliament passed a bill to impose a fine, i.e., an administrative punishment, on restaurants and other establishments that fail to comply with requests to shorten their opening hours and on COVID-19 patients that refuse to be hospitalized. However, as highlighted in numerous studies (e.g., Goolsbee & Syverson, 2021), the direct effects of legally binding intervention policies in the United States and Europe have been limited. Examining whether the decision to strengthen legally binding interventions is appropriate therefore is an important issue for the future.

Finally, we would like to mention an issue that we were unable to fully explore in this study. That is, while we focused on people’s fear as one of the reasons for changes in behavior, another possible reason, as highlighted by Alfaro et al. (2020) and others, is altruism. According to estimates by Bethune & Korinek (2020), due to infection externalities the cost of infection to an individual is only one-third of the cost to society. In other words, avoiding getting infected generates three times the benefit to society as a whole. To achieve this benefit, altruism is indispensable. A key research question for the future therefore concerns the role of altruism during the current and future pandemics, such as the extent to which changes in behavior during the current pandemic were caused by altruism and how changes in behavior due to altruism are linked people’s values and beliefs in normal times.

## Data Availability

The data used in this paper is available at https://doi.org/10.6084/m9.figshare.14658753.v1

## Appendix

See Appendix Figs. 9, 10

## Footnotes

1 Specifically, Ahorsu et al.’s (2020) “Fear of COVID-19 Scale” is based on responses on a five-item scale ranging from “strongly disagree” to “strongly agree” to seven statements such as “It makes me uncomfortable to think about coronavirus-19,” “I am afraid of losing my life because of coronavirus-19,” and “When watching news and stories about coronavirus-19 on social media, I become nervous or anxious,” which were then aggregated to create the fear scale.

2 While beyond the scope of this study, it would be interesting to examine the individual impact of the different requests on the stay-at-home measure. An empirical study that does focus on the impact of a single measure is that by Takaku & Yokoyama (2021), which examines the effect of the closure of schools, carefully isolating it from the effects of other measures simultaneously taken by the central and local governments.

3 As explained earlier, the values of $m_{rt}$ and $n_{rt}$ are obtained by transforming the number of infections using the arcsinh transformation. The coefficients on these two variables, $\beta _{1a}$ and $\beta _{2a}$ indicate that if the number of new infections in a prefecture increases by, for example, 1%, the stay-at-home measure increases by $0.01\times \beta _{1a}$ percentage points. For more details on the arcsinh transformation, see Bellemare & Wichman (2020).

4 For details, see https://mobaku.jp/.

5 There are two possible ways to define the date of infection: the date a person actually contracted the virus, and the date infection with the virus is confirmed. However, since the infected person may not necessarily know for certain when and where they contracted the virus, the date of infection is often impossible to determine. On the other hand, the date of a test result is clearly recorded. Since the number of infections reported in newspapers and on TV is based on the date of the test result, it is likely that people’s decision-making on whether to leave their home is affected by the number of infections based on the date of the test result.

6 See https://www3.nhk.or.jp/news/special/coronavirus/data/. Persons for which the prefecture of residence is unknown or who reside abroad are excluded from the sample.

7 For details, see https://www.mext.go.jp/content/20200304-mxt_kouhou02-000004520_1.pdf (in Japanese).

8 Details of which activities were restricted differed by prefecture. For details, see https://www3.nhk.or.jp/news/special/coronavirus/tokyo/ (in Japanese).

9 The coefficient on the number of new infections fell substantially in mid-August. This was likely due to the fact that residents in urban areas with relatively high infection numbers went out during the Obon holidays. The coefficient on the number of new infections also fell around March 20, which may be due to an increase in outings for cherry blossom viewing.

10 The pattern for minors—i.e., those aged 15–19—differs from that for those in their 20s, and the coefficient for this age group is larger than that for the other age groups even on weekends and public holidays. This suggests that parents, schools, and cram schools play a substantial part in minors’ decision-making about whether to go out or not.

11 In this context, it is worth noting that Bordalo et al. (2020), based on a survey of 1,500 Americans, suggest that for the young the current COVID-19 crisis represents a “disease and death” shock that was particularly surprising and that the young tended to have a higher assessment of the risks associated with the pandemic (risk of infection, risk of serious illness if infected, and risk of death if infected). Applied to Japan, this finding suggests that the risk perceptions of the young may have changed substantially as a result of the declaration of a state of emergency.

12 Alfaro et al. (2020) cite fear and altruism as reasons for changes in people’s behavior. The former is the fear of becoming infected, while the latter is the concern that being infected oneself may endanger those around one. For instance, people wear masks to protect themselves as well as others (see Cheng et al. 2020). Fear is thought to be stronger in the old, who are at a higher risk of serious illness and death. In contrast, there are no a priori reasons to believe that there is a large difference in altruism across age groups. Therefore, if changes in behavior originate from altruism, changes in people’s behavior when they are exposed to information about the pandemic should be unrelated to age.

13 Note that while the regression analysis in this study is based on the assumption that the effect of government intervention on the stay-at-home measure is identical across prefectures, it could potentially be heterogeneous. A task for the future is to check the robustness of our results explicitly taking the possibility of such heterogeneity into account.

14 See, e.g., Kushner (2020) for details.
